# Breast cancer screening participation in women using mental health services in NSW, Australia: a population study

**DOI:** 10.1007/s00127-023-02509-w

**Published:** 2023-06-12

**Authors:** Chris Lambeth, Philip Burgess, Jackie Curtis, David Currow, Grant Sara

**Affiliations:** 1 System Information and Analytics Branch, NSW Ministry of Health, St Leonards, Australia; 2 Biostatistics Training Program, NSW Ministry of Health, St Leonards, Australia; 3 School of Public Health, University of Queensland, Brisbane, Australia; 4 Faculty of Medicine and Health, University of NSW, Kensington, Australia; 5 Faculty of Science, Medicine and Health, University of Wollongong, Wollongong, Australia; 6 Faculty of Medicine and Health, University of Sydney, St Leonards, Australia

**Keywords:** Breast cancer, Screening, Mammography, Mental illness, Schizophrenia, Depression

## Abstract

**Purpose:**

Population screening programs have contributed to reduced breast cancer mortality, but disadvantaged or vulnerable groups may not have shared these improvements. In North American and European studies, women living with mental health conditions have reduced breast screening rates. There are no current Australasian data to support health system planning and improvement strategies.

**Methods:**

The New South Wales (NSW) BreastScreen program offers free screening to NSW women aged 50–74. We compared 2-year breast screening rates for mental health service users (*n* = 33,951) and other NSW women (*n* = 1,051,495) in this target age range, after standardisation for age, socioeconomic status and region of residence. Mental health service contacts were identified through linkage to hospital and community mental health data.

**Results:**

Only 30.3% of mental health service users participated in breast screening, compared with 52.7% of other NSW women (crude incidence rate ratio 0.57, 95% CI 0.56–0.59). Standardisation for age, socioeconomic disadvantage or rural residence did not alter this screening gap. Around 7000 fewer women received screening than would be expected from comparable population rates. Screening gaps were largest in women over 60 and in socioeconomically advantaged areas. Women with severe or persistent mental illness had slightly higher screening rates than other mental health service users.

**Conclusions:**

Low breast cancer screening participation rates for NSW mental health service users suggest significant risk of later detection, possibly leading to more extensive treatment and premature mortality. Focussed strategies are needed to support greater breast screening participation for NSW women who use mental health services.

**Supplementary Information:**

The online version contains supplementary material available at 10.1007/s00127-023-02509-w.

## Background

Breast cancer is the most common cancer in Australian women, accounting for 28% of reported cancers and 14% of deaths [1]. A national program offers free screening for Australian women aged 50–74. In the 3 decades since its implementation, mortality due to breast cancer has halved in participants, more than twice the reduction seen in the broader population [2]. However, disadvantaged or vulnerable groups may not have shared these improvements [2].

Studies of other cancer types have found that people living with mental illness have reduced participation in cancer screening. A recent meta-analysis [3] estimated an odds ratio of 0.76 for participation in any cancer screening, with odds ranging from 0.65 for breast to 0.78 for prostate and 0.89 for cervical cancer screening, but with no reduction in bowel cancer screening (OR 1.02). This may contribute to people with mental health conditions having more advanced cancer at detection, less effective cancer care and increased case fatality rates [4–7]. Women living with psychotic or substance use disorders have been found to have particularly low cancer screening participation rates, with odds of screening as low as 0.5 compared to reference populations [8–10]. However, there are gaps and inconsistencies in the evidence regarding breast cancer screening. Many studies have examined small clinical cohorts. Some population studies use self-report or symptom scores from population surveys to define mental illness [11–13]. Only a handful of studies have examined large, population-wide cohorts of women with diverse mental health diagnoses obtained from clinical registry or claims data [14–16]. These studies find that in North American [9, 14, 16, 17] or European health systems [12, 15, 18], women with mental health conditions are 20–40% less likely to participate in breast cancer screening. There are no current data on this issue from Australasia or South-East Asia. Cultural and health system factors influence breast screening participation rates [19, 20], and therefore, local evidence is required to support health system planning and improvement strategies.

This study aims to describe the rate of BreastScreen participation in women aged 50 to 74 who have had contact with specialist mental health services in New South Wales (NSW), Australia, and compare this to rates in the general population after adjusting for age, socioeconomic status and rurality. The study has been conducted as part of the Mental Health Living Longer project [21]. This is an ongoing, population-wide data linkage, undertaken by the NSW Ministry of Health, focussed on understanding and reducing premature mortality in people using mental health services.

## Methods

### Setting and context

BreastScreen Australia is a joint state and federal government initiative providing free mammograms to women aged 40 and over. Women aged 50–74 years are invited by letter to have a screening mammogram every 2 years. Initial invitations are made by letter, and invitations for repeat screening are made by email, letter or text message as nominated by the individual. A follow-up invitation is sent if screening is not booked within 6 weeks of initial invitation. Bookings for screening can be made on-line or via a national free-call phone number. In NSW, screening occurs at 50 fixed screening sites, or via 15 mobile screening vans which visit approximately 200 locations in rural and regional NSW, annually for most sites or biennially for some more remote sites. The BreastScreen NSW data collection, administered by Cancer Institute NSW, records demographics of the individual, the date and result of mammogram screening, and the results of other diagnostic tests such as histopathology following biopsy.

Mental health care in NSW is mainly government funded or subsidised. Approximately, two-thirds of hospital admissions for mental health care occur in state-funded (public) hospitals. Free public community mental health services provide emergency and acute community mental health care, and community care for people with severe or enduring mental health conditions. Commonwealth-government subsidised, and privately funded services provide primary mental health care and office-based specialist care.

### Study design and data sources

**Fig. 1:**
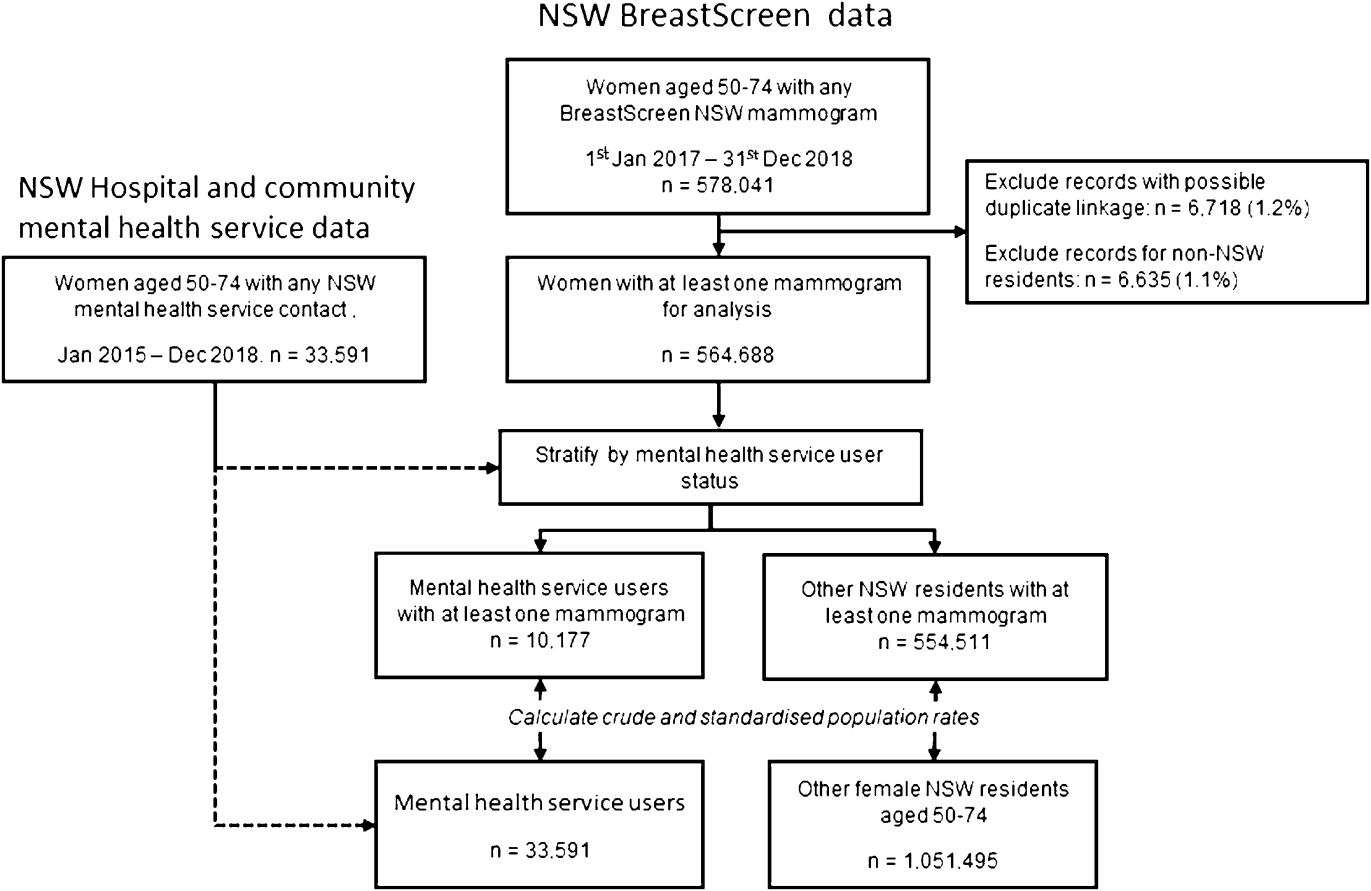
Study flowchart and participant numbers

We used NSW BreastScreen data to estimate breast screening rates for NSW women aged 50–74 years at the time of screening, from 1 Jan 2017 to 30 Dec 2018 (See Fig. 1). We excluded (i) non-NSW residents and (ii) a small number of women with duplicate linkages between BreastScreen and NSW Health data.

We defined mental health service users by linkage to data from NSW public community mental health services (NSW Mental Health Ambulatory Data Collection) and NSW public and private hospitals (NSW Admitted Patient Data Collection). Mental health service use was defined as at least one contact with mental health services in the 2 years prior to or during the study period (2015–2018), and prior to mammographic screening. In-scope contacts included at least one clinical contact with a NSW Community Mental Health service, a hospital admission with at least one inpatient day in a designated mental health unit, or a hospital admission with a mental health diagnosis as the primary reason for hospital admission.

### Statistical analysis

Data assembly and statistical analysis were conducted in SAS Enterprise Guide v7.15. Screening participation rates were calculated as the percentage of women who participated in screening with BreastScreen NSW over a 2-year period, aligning with the 2-year recommended screening interval. The population denominator for rate calculation was the NSW estimated resident female population aged 50–74 at the mid-point of the study period. Adjusted rates were calculated by direct standardisation to NSW population distributions using the SAS STDRATE procedure [22]. Rates were standardised for age group, rural location and socioeconomic disadvantage. Socioeconomic disadvantage was estimated from the person’s area of residence, using the Australian Bureau of Statistics (ABS) Index of Relative Socioeconomic Disadvantage (IRSD) (Australian Bureau of Statistics 2006). This index is calculated using census-derived variables measuring income, government welfare support, education, home ownership, employment, household structure and English language proficiency from postcodes. Disadvantage was analysed as an ordinal variable, using ABS quintiles to split regions into five groupings of roughly equal population size.

We defined a sub-group of mental health service users with “Serious or Persistent Mental Illness” (SPMI), as indicated by (i) at least one recorded diagnosis of a psychotic disorder (including schizophrenia, schizoaffective disorder, brief or atypical psychoses, mania, bipolar disorder or psychotic depression), or (ii) a total span of mental health treatment (time from first hospital or community mental health contact to last hospital or community mental health contact in the study period) of more than two years. For inclusion in the mental health service user group, mental health service contact had to occur before the first breast screening record. However, in that group, diagnoses or contacts occurring throughout the whole study period, including after breast screening participation, were also examined for defining the SPMI sub-group. Diagnoses in NSW Health data are recorded using the Australian modification of the International Classification of Diseases, Version 10 (ICD-10) [23]. Hospital diagnoses are coded by trained health information managers. Community diagnoses are recorded directly by clinicians in diverse electronic medical record applications. To compare participation rates for the SPMI sub-group to other mental health service users, we used a Poisson regression model with robust standard errors [24], using screening status as the binary outcome variable and SPMI group membership as a predictor variable, either alone (unadjusted model) or after adjustment for age, socioeconomic status and rurality (adjusted model).

Sensitivity analysis was conducted to examine for possible effects of people living near state borders who may have had screening in neighbouring communities interstate and, therefore, not be included in NSW data. Screening rates and rate ratios were re-calculated after exclusion of residents of local government areas (LGA) adjacent to state borders with Queensland (Tweed, Ballina, Byron LGAs) and Victoria (Albury, Murrumbidgee LGAs).

## Results

After exclusion of non-NSW residents and a small number of records with duplicate linkage, 564 688 women with at least one mammogram during the study period were included in the study. In the study period, there were 33 591 women aged 50–74 who had contact with NSW mental health services. Compared to other NSW women of the same age (*n* = 1,051,495), mental health service users were more likely to be aged under 60, and slightly more likely to live in disadvantaged or regional and rural areas (Table 1). Forty percent (40%) of mental health service users met the operational definition for severe or persistent mental illness.

**Table 1:** Cohort description. Characteristics of NSW population (women aged 50–74) and NSW BreastScreen participants. Mental health (MH) service users compared to other NSW residents

	NSW population, women aged 50–74 years		BreastScreen participants	
	MH service users (%)	Other NSW residents *N* (%)	MH service users *N* (%)	Other NSW residents *N* (%)
Total	33,591	1,051,495	10,177	554,511
Age group^1^				
50–54	9460 (28.2)	243,720 (23.2)	2619 (25.7)	113,361 (20.4)
55–59	8443 (25.1)	237,312 (22.6)	2546 (25.0)	121,575 (21.9)
60–64	6252 (18.6)	217,878 (20.7)	2125 (20.9)	122,068 (22.0)
65–69	4951 (14.7)	195,793 (18.6)	1667 (16.4)	110,856 (20.0)
70–74	4485 (13.4)	156,792 (14.9)	1220 (12.0)	86,651 (15.6)
Rurality area^2^				
Major cities	23,656 (68.1)	738,722 (70.7)	6608 (65.0)	380,369 (68.7)
Inner regional	8042 (24.2)	232,429 (22.2)	2777 (26.3)	135,183 (24.4)
Outer regional	2304 (6.9)	70,787 (6.8)	791 (7.8)	35,941 (6.5)
Remote/very remote	277 (0.8)	3498 (0.3)	93 (0.9)	1961 (0.4)
Socioeconomic disadvantage^3^				
1st (most)	6881 (20.7)	205,536 (19.7)	1980 (19.5)	87,492 (15.8)
2nd	6525 (19.6)	227,043 (21.7)	2168 (21.3)	105,761 (19.1)
3rd	7972 (24.0)	203,811 (19.5)	2427 (23.9)	119,323 (21.6)
4th	6502 (19.5)	197,248 (18.9)	1899 (18.7)	114,306 (20.7)
5th (least)	5399 (16.2)	211,763 (20.3)	1695 (16.7)	126,572 (22.9)
Severe and persistent mental illness (SPMI)				
SPMI	13,343 (39.7)	–	4145 (40.7)	–
Other	20,248 (60.3)	–	6032 (59.3)	–

Population proportions compared using Chi-squared test

^1^Age: *χ*^2^ (df4) = 829.5, Prob < 0.0005

^2^Rurality: *χ*^2^(df3) = 248.2, Prob < 0.0005

^3^Socioeconomic disadvantage: *χ*^2^ (df4) = 678.2, Prob < 0.0005

Only 30.3% of mental health service users participated in breast screening in the study period, compared with 52.7% of other NSW women (IRR 0.57, 95% CI 0.56–0.59) (Table 2). Standardisation for age alone or in combination with socioeconomic status or rurality produced only negligible changes in estimated rates or incidence rate ratios.

**Table 2:** Breast cancer screening rates in women aged 50–74 using mental health services, compared to other NSW women of the same age

	MH service users percent (95% CI)	Other NSW residents percent (95% CI)	Rate ratio (95% CI)
Crude screening rate	30.3 (29.7–30.9)	52.7 (52.6–52.9)	0.57 (0.56–0.59)
Age standardised rate	30.6 (30.0–31.2)	52.7 (52.6–52.9)	0.58 (0.57–0.59)
Age + disadvantage standardised rate	31.0 (30.4–31.6)	52.9 (52.8–53.1)	0.59 (0.57–0.60)
Age + rurality standardised rate	30.8 (30.2–31.4)	52.9 (52.8–53.1)	0.58 (0.57–0.59)

**Fig. 2:**
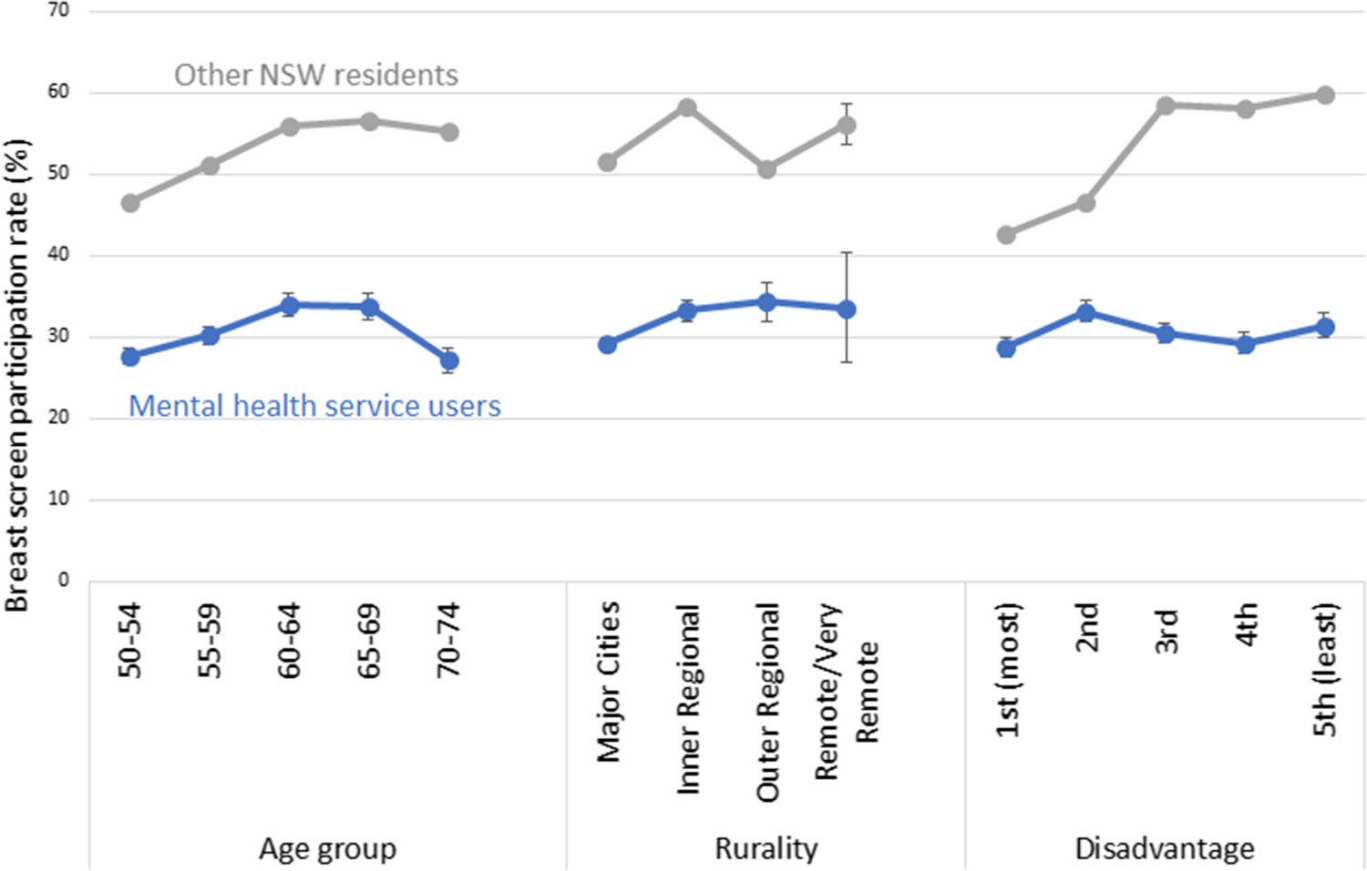
Breast screening participation rates, by age group, rurality and socioeconomic disadvantage, comparing NSW mental health service users aged 50–74 to other NSW women of the same age

Breast screening participation rates differed for different strata of age, socioeconomic region and rurality (Fig. 2, Online Resource 1). For the broader population, screening participation rates increase with age and with increasing socioeconomic status. In women with recent mental health service contact, these gradients were much less evident. As a result, the greatest gaps in screening participation (lowest incidence rate ratios) occurred where population screening rates were highest, in women aged 65 and over and in least disadvantaged localities. The relationship between rural location and screening participation was less clear in both mental health service users and the broader population, with much greater uncertainty of estimates for remote and very remote regions due to much smaller populations.

In sub-group analyses (Table 3), women with severe or persistent mental illness had a slightly higher screening rate than other mental health service users after adjusting for age, socioeconomic status, and rurality (adjusted Rate Ratio 1.05, 95% CI 1.02–1.09).

**Table 3:** MH sub-group analysis, adjusted for age, sex, disadvantage

	Screening rate (95% CI)	Unadjusted RR (95% CI)	Adjusted RR (95% CI)
Other mental health service users	29.8 (29.0–30.5)	1.00	1.00
Severe and persistent mental illness (SPMI)	31.1 (30.1–32.0)	1.04 (1.01–1.08)	1.05 (1.02–1.09)

Reference category is Other Mental Health Service Users

In sensitivity analysis, exclusion of LGAs adjacent to state borders had almost no effect on screening rates and rate ratios. Revised standardised rates were 30.7% in MH service users, 53.5% in other NSW women, and aIRR 0.57 (95% CI 0.56–0.59).

## Discussion

This study provides the first Australian estimates of breast screening participation in women who use mental health services. We found that in NSW, less than one-third (30.8%) of women who used mental health services participated in breast screening, compared to more than half of other NSW women of the same age. This represents more than 7000 mental health service users who would be screened annually if participation rates matched the broader population. This screening gap has potentially serious impacts on cancer morbidity (from the cancer and the need for more extensive therapy when diagnosed) and mortality. Breast screening may reduce mortality in eligible populations by 15–20%, with one breast cancer death averted for each 500 persons screened [8].

Low BreastScreen participation rates in NSW mental health service users were not explained by being younger or more likely to live in disadvantaged or rural locations. The screening participation rate in NSW mental health service users was more than 10 percentage points lower than recent participation estimates for two recognised priority groups in the NSW population: women born in non-English speaking countries (24-month screening rate 43%), and Aboriginal or Torres Strait Islander women (24-month screening rate 41%) [25]. We found similar relative reductions in screening to those reported for women with psychoses or serious mental illness in US and European studies [8, 9]. This suggests that despite high population recognition and reach, universal screening programs such as BreastScreen NSW need additional strategies to reach disadvantaged and high-risk groups [2], and that women who use mental health services should be considered a priority group for such supplementary strategies.

We found little variation in participation rates in mental health service users across the target age range (50–74) or based on rurality or disadvantage. In the broader population, participation rates increased with age and in less socioeconomically disadvantaged regions. Since these gradients were not present in mental health service users, we found the largest gaps in participation rates in older women (aged 70–74) and in less disadvantaged regions. The absence of usual population gradients suggests there may be additional shared barriers to breast screen participation in women who use mental health services. The few studies examining this issue have highlighted potential health system barriers including physical accessibility, provider attitudes regarding mental illness, concerns about stigma or embarrassment, and lack of integration with general and primary health care [10, 26].

Unexpectedly, we found that women defined as having severe or persistent mental illness had a slightly higher screening participation rate than other mental health service users. There are conflicting findings regarding associations between breast screening rates and specific mental health diagnoses. Meta-analytic reviews find significant heterogeneity and overlapping estimates [8, 9]. It has been argued that evidence for a specific association with psychotic disorders or severe illness is weak, and that reduced screening rates may be pan-diagnostic [10].

Despite ongoing health promotion efforts, breast cancer screening rates in the broader Australian population have been stable over the last 2 decades [2]. There have been efforts to supplement universal screening with risk-based approaches which actively recruit women with personal or family risk factors [27]. Cancer Institute NSW works with community groups to promote screening in Aboriginal women, women from non-English speaking backgrounds, LGBTIQ women and women in prison settings. There is little evidence for specific strategies to improve breast screening in women living with mental ill health, but some studies in this group have identified barriers to breast and cervical cancer screening including shame and stigma, lack of integration of physical and mental health services, and past experiences of sexual trauma [28–30]. To address barriers and improve screening in marginalised groups, services need to engage communities in culturally safe ways and involve them in co-design of solutions [31, 32].

### Limitations

The screening register used in this study includes all participants in a national publicly funded screening program. It does not include women who choose to have mammography privately outside of that program, who are estimated to comprise up to 9.5% of NSW women in this age group. Private mammography is associated with out-of-pocket costs and is more likely in economically advantaged areas. Therefore, its exclusion may bias our results towards underestimating the socioeconomic gradient in screening participation rates, and underestimating the screening gap for mental health service users, particularly in more advantaged areas.

In this study, mental health service use is defined by hospital admissions or contact with public community mental health services. We do not have access to linked data on mental health services in primary care or private specialist settings, and therefore, our findings may not generalise to the larger group of Australian women with more prevalent and/or milder mental health conditions who receive care only in that service sector.

Our findings regarding “serious and persistent mental illness” may reflect limitations in our sub-group definition. Due to limited diagnostic completeness in NSW community mental health data, we also defined this group by a longer span of service contact. A 2-year threshold for illness duration is a component of many definitions of serious or persistent mental illness [33]. However, it is possible that women who maintain contact with mental health services over 2 or more years may also be more likely to engage with other primary health or prevention services. Alternatively, this finding may reflect that, regardless of diagnosis, all women who had hospital admissions for mental health conditions or contact with state operated community mental health teams have serious or potentially disabling illness. Our mental health cohort comprised approximately 2% of the in-scope NSW population, consistent with typical estimates of the prevalence of severe or persistent illness.

## Conclusions

Population breast screening programs reduce breast cancer mortality. In this study, mental health service users had rates of screening even lower than that reported for other disadvantaged groups in the NSW population. Screening rates were low across the target age range, for all regions and levels of socioeconomic disadvantage, and for women with and without severe and persistent mental illness. This suggests that broad strategies are needed to overcome barriers to participation and support mental health service users.

### Supplementary Information

Below is the link to the electronic supplementary material.

Supplementary file1 (DOCX 16 KB)

## Data Availability

Access to NSW Health data is available to researchers only with the specific approval of the NSW Population and Health Services Research Ethics Committee (https://www.cancer.nsw.gov.au/research-and-data/nswpopulation-health-services-research-ethics-com). That approval does not permit sharing of unit record data with other researchers.
