# Supplementary material for: Breast cancer screening participation in women using mental health services in NSW, Australia: a population study
Source: Soc Psychiatry Psychiatr Epidemiol. 2023 Jun 12;59(5):839–46. doi: 10.1007/s00127-023-02509-w (PMC11087311; doi:10.1007/s00127-023-02509-w)
Supplement: Supplementary file 1 — Supplementary file1 (DOCX 16 KB) [file 127_2023_2509_MOESM1_ESM.docx]

# SUPPLEMENTARY INFORMATION

*Online Resource 1. Breast screening participation rates, by age-group, rurality and socioeconomic disadvantage, comparing NSW mental health service users aged 50-74 to other NSW women of the same age (Data for Figure 2).*

|  | MH service users (95% CI) | | Other NSW residents  (95% CI) | | IRR (95% CI) |
| --- | --- | --- | --- | --- | --- |
| Age group |  | |  | |  |
| 50-54 | 27.7 (26.6-28.7) | | 46.5 (46.2-46.8) | | 0.60 (0.57-0.62) |
| 55-59 | 30.2 (29.0-31.3) | | 51.2 (50.9-51.5) | | 0.59 (0.57-0.61) |
| 60-64 | 34.0 (32.5-35.4) | | 56.0 (55.7-56.3) | | 0.61 (0.58-0.63) |
| 65-69 | 33.7 (32.1-35.3) | | 56.6 (56.3-57.0) | | 0.59 (0.57-0.62) |
| 70-74 | 27.2 (25.7-28.7) | | 55.3 (54.9-55.6) | | 0.49 (0.47-0.52) |
| Rurality Area |  | |  | |  |
| Major Cities | 29.2 (28.5-29.9) | | 51.5 (51.3-51.7) | | 0.57 (0.55-0.58) |
| Inner Regional | 33.3 (32.0-34.5) | | 58.2 (57.9-58.5) | | 0.57 (0.55-0.59) |
| Outer Regional | 34.3 (31.9-36.7) | | 50.8 (50.2-51.3) | | 0.68 (0.63-0.73) |
| Remote/Very Remote | 33.6 (26.8-40.4) | | 56.1 (53.6-58.6) | | 0.60 (0.49-0.74) |
| Socioeconomic Disadvantage | |  | |  | |
| 1st (most) | 28.8 (27.5-30.0) | | 42.6 (42.3-42.8) | | 0.68 (0.65-0.71) |
| 2nd | 33.2 (31.8-34.6) | | 46.6 (46.3-46.9) | | 0.71 (0.68-0.74) |
| 3rd | 30.4 (29.2-31.7) | | 58.5 (58.2-58.9) | | 0.52 (0.50-0.54) |
| 4th | 29.2 (27.9-30.5) | | 58.0 (57.6-58.3) | | 0.50 (0.48-0.53) |
| 5th (least) | 31.4 (29.9-32.9) | | 59.8 (59.4-60.1) | | 0.53 (0.50-0.55) |
